# Decoration of viscose fibers with silver nanoparticle-based titanium-organic framework for use in environmental applications

**DOI:** 10.1007/s11356-024-31858-5

**Published:** 2024-01-19

**Authors:** Mohamed Rehan, Ahmed S. Montaser, Mahmoud El-Shahat, Reda M. Abdelhameed

**Affiliations:** 1 Department of Pretreatment and Finishing of Cellulosic-Based Textiles, Textile Research and Technology Institute, National Research Centre, 33 Bohoth Street, Dokki, P.O. Box 12622, Giza, Egypt; 2 Photochemistry Department, Chemical Industries Research Institute, National Research Centre, Scopus Affiliation ID 60014618, 33 EL Buhouth St., Dokki, Giza, 12622 Egypt; 3 Applied Organic Chemistry Department, Chemical Industries Research Institute, National Research Centre, Scopus Affiliation ID 60014618, 33 EL Buhouth St., Dokki, Giza, 12622 Egypt

**Keywords:** Catalytic property; in situ silver nanoparticles, Metal–organic framework (MOF), Sonocatalytic, Sonophotocatalytic, Sulfa drug, Ultrasonic irradiation, Viscose fibers

## Abstract

To effectively remove pharmaceuticals, nitroaromatic compounds, and dyes from wastewater, an efficient multifunctional material was created based on silver nanoparticles (Ag) and MIL-125-NH_2_ (MOF) immobilized on viscose fibers (VF) as a support substrate. Firstly, silver nanoparticles (Ag) were immobilized on the surface of viscose fibers (VF) via in situ synthesis using trisodium citrate (TSC) as a reducing agent to create (VF-Ag). Then, VF and VF-Ag were decorated with the titanium metal–organic framework MIL-125-NH_2_ (MOF) to create VF-MOF and VF-Ag-MOF. The influence of VF-Ag, VF-MOF, and VF-Ag-MOF on the sonocatalytic or sonophotocatalytic degradation of sulfa drugs was investigated. The results show that VF-Ag-MOF showed excellent sonocatalytic and sonophotocatalytic activity towards the degradation of sulfa drugs compared to VF-Ag and VF-MOF. Furthermore, sonophotodegradation showed a dramatic enhancement in the efficiency of degradation of sulfa drugs compared to sonodegradation. The sonophotodegradation degradation percentage of sulfanilamide, sulfadiazine, and sulfamethazine drugs in the presence of VF-Ag-MOF was 65, 90, and 95 after 45 min of ultrasonic and visible light irradiation. The catalytic activity of VF-Ag, VF-MOF, and VF-Ag-MOF was evaluated through the conversion of p-nitrophenol (4-NP) to p-aminophenol (4-AP). The results demonstrate that VF-Ag-MOF had the highest catalytic activity, followed by VF-Ag and VF-MOF. The conversion percentage of 4-NP to 4-AP was 69%. The catalytic or photocatalytic effects of VF-Ag, VF-MOF, and VF-Ag-MOF on the elimination of methylene blue (MB) dye were investigated. The results demonstrate that VF-Ag-MOF showed high efficiency in removing the MB dye through the reduction (65%) or photodegradation (71%) after 60 min. VF-Ag-MOF composites structure–activity relationships represent that doping within silver NPs enhanced the photocatalytic activity of MIL-125-NH_2_, which could be explained as follows: (i) Due to the formation of a Schottky barrier at the junction between MIL-125-NH_2_ and Ag NPs, the photogenerated electrons in the conduction band of MIL-125-NH_2_ were supposed to be quickly transferred to the valence band of the Ag NPs, and subsequently, the electrons were transferred to the conduction band of Ag NPs. This considerable electron transferring process, which is reported as Z scheme heterojunction, can efficiently suppress the recombination of electron/hole pairs in VF-Ag-MIL-125-NH_2_ composites. (ii) Sufficient separation between the photogenerated charge carriers (holes and electrons) and avoiding their recombination enhanced the photocatalytic activity of composites.

## Introduction

Water pollution remains the most significant environmental problem worldwide. Organic contaminants such as pharmaceuticals, nitro-aromatic compounds, and dyes are considered the primary sources of water pollution (Murtaza et al. 2023). These pollutants are extremely hazardous and may harm aquatic life as well as human health. Hence, the removal of these pollutants is paramount in the assessment of contamination risks and the minimization of potential health hazards (Rehan and Elhaddad 2024). There are certain challenges in this sector, despite the numerous studies on photocatalysis or catalytic for destroying pharmaceuticals (Bagheri et al. 2017, Majumdar and Pal 2020, Pham et al. 2023, Sawunyama et al. 2023), nitroaromatic compounds (Aswathy et al. 2023; Beiranvand et al. 2023; Shahzaib et al. 2023), and dyes (Guo et al. 2023; Rafiq et al. 2021; Saeed et al. 2022). The majority of the literature focused on removing only one sort of contaminant at a time. Based on the aforementioned, more effort must be placed into creating effective methods to remove pharmaceuticals, nitroaromatic compounds, and dyes using a single material to reduce contamination concerns and associated health hazards. The advanced oxidation process has emerged as a promising strategy for the degradation of organic contaminants. Sonocatalysis and photocatalysis are considered two methods of the advanced oxidation process that can be used to remove and degrade organic contaminants. The sonocatalysis process uses a semiconductor in the presence of ultrasound waves but without the presence of light irradiation. The photocatalysis process uses a semiconductor in the presence of light irradiation. Recently, sonophotocatalysis is a new method of the advanced oxidation process. Sonophotocatalysis is a combination of sonocatalysis and photocatalysis. Sonophotocatalysis is the integration between ultrasound waves, light irradiation, and a semiconductor photocatalyst (Joseph et al. 2009). Metal–organic frameworks (MOFs) have recently emerged as innovative photocatalysts because of their distinctive high surface area; well-ordered porosity structure, simple modification, and metal–organic frameworks (MOFs) are an intriguing class of porous materials created by combining a metal oxo-cluster and an organic linker (Qiu et al. 2018).

MOFs have garnered considerable attention for use in various fields especially in environmental applications to remove organic contaminants from wastewater (Poonia et al. 2023; Ramalingam et al. 2022; Sriram et al. 2022; Yan et al. 2022). Adsorption (Amenaghawon et al. 2023; Du et al. 2021; Emam et al. 2020; Russo et al. 2020; Younes et al. 2022) or photocatalytic are two techniques for removing pollutants from wastewater using MOFs (Abdelhameed et al. 2023; Du et al. 2021; Liu et al. 2023b; Russo et al. 2020; Xia et al. 2021). Lots of modified strategies have emerged with the rapid development of photocatalysts based on MOFs, e.g., decoration of metal centers or linkers, combination with semiconductors, metal nanoparticle (NP) loading, sensitization, and pyrolyzation. Therefore, metal NP loading is regarded as one of the most efficient approaches owing to their large surface area, more exposure of reaction sites, and the high density of the coordination unsaturated sites. However, metal NPs tend to migrate and aggregate due to their high surface energy. Metal NPs loading on MOFs could divide into the following ways: dispersion on the surface (Liang et al. 2015a, 2015b) and encapsulation into the cavities (Abdelhameed et al. 2023; Chen et al. 2014; Sun et al. 2018; Wang et al. 2018). Titanium metal–organic framework (Ti-MOF) has recently received a lot of interest in the field of environmental remediation (Abdelhameed et al. 2022; Chen et al. 2020; Zhu et al. 2018). MIL-125-NH_2_, one of the Ti-based MOFs, has a relatively wide surface area and may function as a typical adsorption medium with photocatalytic activity (Abdelhameed et al. 2021). Therefore, MIL-125-NH_2_ has become a particularly intriguing material for environmental applications (Abdelhameed et al. 2020a, Abdelhameed and El-Shahat 2019, El-Shahat et al. 2020, Mubarak et al. 2022, Yao et al. 2022). Encapsulating metal NPs into the cavities of MOFs will prevent the loss of NPs efficiently and verify the excellent recycling test, but the specific window size limits its general applicability. Therefore, many reports were focused on the metal NP surface loading. Shen et al. synthesized M/MIL-125-NH_2_ composites via a redox reaction between the reductive MIL-125 with Ti^3+^ and oxidative metal salt precursors for the photocatalytic process (Shen et al. 2015). To enhance the photocatalytic properties of MIL-125-NH_2_, the researchers explored new methods by the doping of noble nanoparticles or metal oxides on MIL-125-NH_2_ surface (Abdelhameed et al. 2020b, 2018b; Emam et al. 2019; Kaur et al. 2022; Luo et al. 2020; Muelas-Ramos et al. 2021; Sheng et al. 2023; Yue et al. 2021). Silver nanoparticles (Ag NPs) are commonly used for doping the titanium-based MOFs to considerably increase photocatalysis (Cheng et al. 2022; Liu et al. 2023a, 2021; Qiu et al. 2022; Sahoo et al. 2022). Ag NPs enhance the photocatalytic properties of MIL-125-NH_2_ by increasing visible light absorption due to their surface plasmon resonance (SPR). Furthermore, Ag NPs can operate as efficient electron traps to limit photoinduced electron–hole (e^−^/h^+^) recombination, resulting in decreased recombination (Che et al. 2023; Liao et al. 2023; Zhu et al. 2015). Furthermore, Ag NPs show remarkable catalytic properties, and they have been used as catalysts to improve the performance of catalyst reactions (Nouri et al. 2020; Rehan et al. 2020). There are some problems with the applications of MOF and nanoparticle-MOF-based MOF materials in environmental fields such as separating, recovering, and recyclability of MOF. Thus, the immobilization of MOF on various substrates can play an important role in adopting an effective and easy recovery and multi-reuse capability (Abdelhameed and El-Shahat 2023, Abdelhameed and Emam 2019, 2022, Abdelhameed et al. 2018a; Emam et al. 2021). Viscose fibers (VF), a form of regenerated cellulosic fiber, have sparked extensive interest in the textile and chemical sectors due to their flexibility, excellent moisture absorption, ion exchange capacity, low cost, non-toxicity, and biodegradability. Viscose fibers can be introduced as a support and carrier substrate and can provide an easy substrate for use and removal after use in environmental fields. Viscose fibers are regenerated cellulose fibers that can be produced with wet-spun cellulose solutions prepared from cotton linter, bamboo, or wood. Viscose fiber is a recently developed support and carrier substrate that may be easily used and removed from wastewater treatment systems (Jiang and Zhou 2021, Liu et al. 2020a, 2020b; Rehan et al. 2019).

Based on the aforementioned and to address the above issues, the fundamental goal of the current study is to design a cost-effective strategy for wastewater treatment by investigating the synergetic effect of noble nanoparticles (Ag) and MIL-125-NH_2_ (MOF) supported on the surface of the viscose fibers (VF) for its use in removing the different pollutants. Thus, the novelty of the current study is to evaluate the efficiency of the as-developed catalyst based on the Ag-MOF immobilized on the VF surface in challenging environmental applications by studying the removal of different organic contaminations from wastewater through different mechanisms. Most of the literature only described one material to eliminate one kind of pollution at a time. The current study is interested in the removal of different pollutants including sulfa drugs, nitroaromatic compounds, and dyes from water through different mechanisms using hybrids containing silver nanoparticles and MOF immobilized on the viscose fibers.

## Experimental

### Materials and chemicals

Viscose fibers (VF) were obtained from Lenzing, Austria. Silver nitrate (Ag NO_3_) was obtained from Panreac, Barcelona, Spain. Trisodium citrate (TSC) was supplied by Sigma-Aldrich (Germany). Ethanol was obtained from Sigma-Aldrich, Germany. Titanium isopropoxide (C_12_H_28_O_5_Ti, 97%,), 2-aminoterephthalic acid (C_8_H_7_NO_4_, 99%), and sulfadiazine (99.0–101.0%) were obtained from Sigma-Aldrich, Germany. Sulphanilamide (BDH laboratory reagent) and sulfamethazine were obtained from Spectrum Chemical MFG Corp., England.

### Simultaneous synthesis and incorporation of silver nanoparticles (Ag) on the surface of viscose fibers surface (VF) using TSC

Five grams of VF was submerged in a solution of silver nitrate (500 ppm) for 30 min at room temperature using a liquor ratio of 1:50. Thereafter, a 25 mL aqueous solution of (2%) TSC was added dropwise to the mixture. The resulting mixture was heated in the water bath under shaking (200 rpm) at 90 °C for 30 min. After that, VF-Ag samples were taken out, squeezed, and rinsed under running water before being dried at room temperature before being analyzed and applied. The absorbance of the supernatant was measured after the end of the reaction.

### Synthesis of titanium metal–organic framework MIL-125-NH_2_ (MOF)

Solvothermal synthesis was used to create MIL-125-NH_2_ (MOF): 30 mL of DMF was used to dissolve 1 mL of titanium isopropoxide and 1.025 g of 2-aminoterephthalic acid. A 150 mL Teflon-lined stainless steel autoclave was filled with the uniform solution, and it was heated at 150 °C for 18 h. The finished goods were dried in an oven at 70 °C after being washed three times with DMF and ethanol.

### Decoration of VF and VF-Ag with MOF

To decorate VF and VF-Ag with MOF, 50 mg of MOF was dispersed in 100 mL of DMF, before immersing VF and VF-Ag in the solution for 1 h at 50 °C with continual stirring. The decorated VF and VF-Ag were then taken out and dried for 2 h at 80 °C. The decorated VF and VF-Ag were then repeatedly rinsed with deionized water and dried at room temperature to obtain VF-MOF and VF-Ag-MOF.

### Characterization and measurement

The formation of the Ag NPs was examined by a UV–visible spectrophotometer (UV–visible multi-channel spectrophotometer (T80 UV/VIS, *d* = 10 mm, PG Instruments Ltd, Japan) from 300 to 600 nm. The morphology of Ag and MOF was evaluated by transmission electron microscopic (TEM). TEM image was taken at an accelerating voltage of 120 kV (Tecnai G2 Spirit, FEI Company, USA). The morphology of MOF (powder), VF, VF-Ag, VF-MOF, and VF-Ag-MOF surfaces was evaluated by scanning electron microscopy (SEM) and dispersive X-ray spectroscopy (EDX) (ZEISS LEO 1530 Gemini Optics Lens scanning electron microscopy (SEM) with 30 kV scanning voltages was employed to observe the morphologies of untreated and treated fabrics. Zeiss LEO 438 VP with Oxford Instruments EDX with INCA software system. EDX measurement conditions, 20 kV accelerating voltage, 21 mm working distance, 1 nA sample). The FTIR spectroscopy (PerkinElmer Co., Ltd., MA, USA) analyzed the surface of MOF, VF, VF-Ag, VF-MOF, and VF-Ag-MOF. X-ray diffraction (XRD) was used for phase identification and crystal structural analysis for MOF (powder) (PANalytical X’pert PRO PW 3040/60 (Netherlands) X-ray diffraction fitted with a Cu Kα (*λ* = 0.154 nm) radiation source in range 2*θ* = (10–80°)).

### Sulfa drugs removal: sonocatalytic degradation and sonophotocatalytic degradation

In this study, sulfa drug removal through sonocatalytic or sonophotocatalytic degradation was investigated. In the photoreactor with 50 mL of sulfanilamide, sulfadiazine, or sulfamethazine (100 ppm), 15 mg of VF, VF-Ag, VF-MOF, or VF-Ag-MOF was added. The photoreactor was magnetically stirred and kept under or without visible light (LED visible lamp with 12 W and light intensity ca. 0.303 W m^−2^ in the range 315–400). Every 5 min, a sample from the photocatalytic reaction was taken and examined for sulfa drug content using a UV–vis spectrometer. The sulfa drug degradation percentage was determined by comparing the absorption intensity (*I*) over time (*t*) to the initial absorption intensity (*I*^0^).

### Evaluation of catalytic property (conversion of 4-NP to 4-AP)

In a beaker, 20 mL of p-nitro phenol (1 mM) was mixed with 5 mL of freshly prepared NaBH_4_ solution (0.3 M). The color of the solution changed from light yellow to yellowish green. Then, 20 mg of VF, VF-Ag, VF-MOF, or VF-Ag-MOF were immersed in this solution. The conversion of p-nitro phenol to p-aminophenol was evaluated by using a UV–vis spectrophotometer after 60 min.

### Evaluation removal of methylene blue (MB) dye (catalytic vers photodegradation)

In the catalytic experiment, 50 mL of 5 mg/L of MB was mixed with 5 mL of freshly prepared NaBH_4_ solution (0.3 M). Then, 20 mg of VF, VF-Ag, VF-MOF, or VF-Ag-MOF were immersed in this solution and continuously stirred at room temperature. In the photodegradation experiment, 20 mg of VF, VF-Ag, VF-MOF, or VF-Ag-MOF were immersed in 50 mL of 5 mg/L of MB and magnetically stirred for 15 min in the dark to achieve an adsorption–desorption equilibrium. Then, it was continuously stirred and exposed to visible light irradiation (LED visible lamp with 12 W and light intensity ca. 0.303 W m^2^ in the range 315–400) (Rehan et al. 2018). In both experiments, the reduction or photodegradation of MB was evaluated after 60 min by measuring the decreasing absorption at a wavelength of 665 nm using a UV–vis spectrophotometer through the following equation:

$$\mathrm C\mathrm a\mathrm t\mathrm a\mathrm l\mathrm y\mathrm t\mathrm i\mathrm c\;\mathrm{or}\;\mathrm p\mathrm h\mathrm o\mathrm t\mathrm o\mathrm d\mathrm e\mathrm g\mathrm r\mathrm a\mathrm d\mathrm a\mathrm t\mathrm i\mathrm o\mathrm n\;\%=\frac{C_0-C_t}{C_0}\times100$$

where *C*_0_ denotes initial dye concentration and *C*_*t*_ denotes dye concentration after 60 min.

### Statistical analysis

The measurements were performed in triplicate. Experiment results were calculated with an average of 3 independent measurements. Every one of the as-shown figures was created with the Origin 8 application.

## Results and discussion

### Characterization of Ag, MOF (powder), VF, VF-Ag, and VF-Ag-MOF

**Fig. 1:**
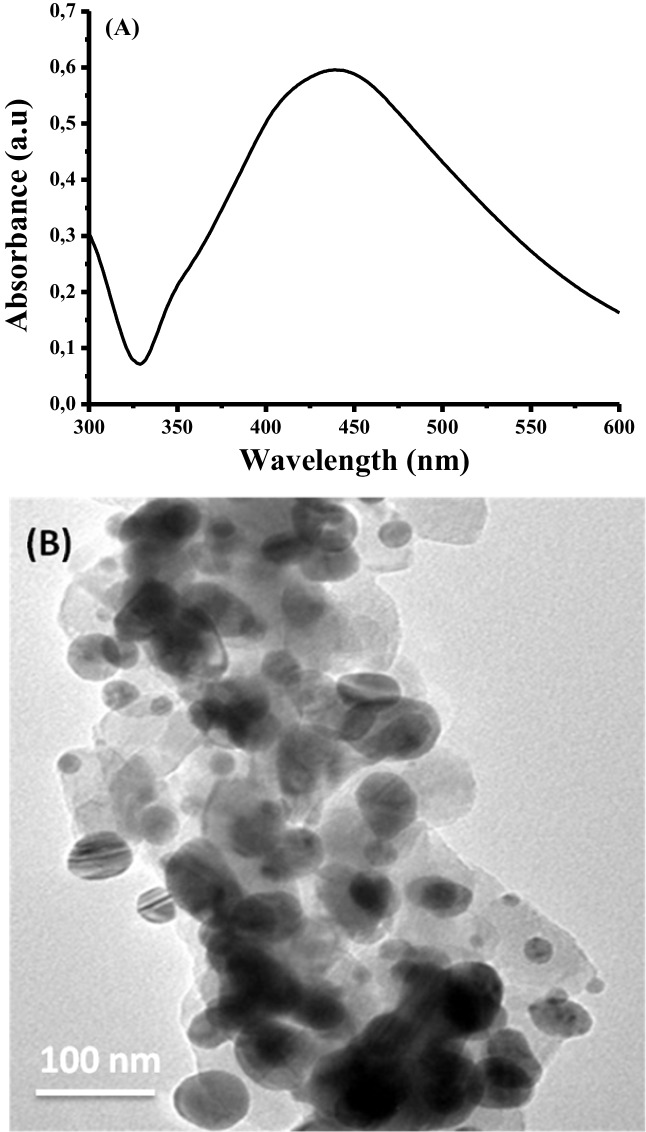
UV–vis absorbance spectra (**A**) and TEM (**B**) of silver nanoparticles

The formation of silver nanoparticles was confirmed by the appearance of an absorption peak at 450 nm in the UV–vis spectroscopy of silver nanoparticles (Fig. 1A), which was attributed to the LSPR properties of the spherical Ag (Emam et al. 2014). The TEM image of silver nanoparticles (Fig. 1B) reveals that the formed silver nanoparticles were almost spherical in shape, homogeneous, and monodisperse with some agglomeration.

**Fig. 2:**
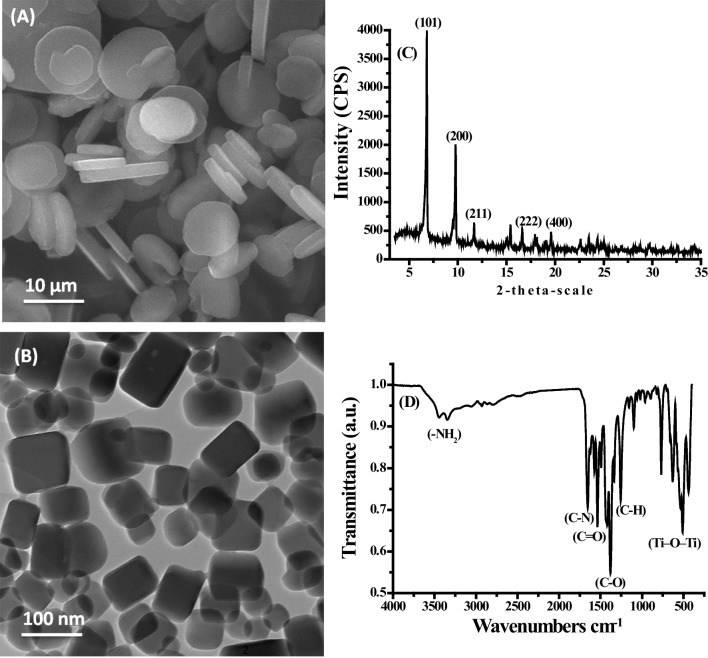
SEM (**A**), TEM (**B**), XRD (**C**), and FTIR (**D**) of MIL-125-NH_2_

The SEM image (Fig. 2A) and TEM image (Fig. 2B) of MIL-125-NH_2_ (MOF) demonstrate that the powder has a smooth surface, rounded corners, and a disk-like morphology. The XRPD pattern of MIL-125-NH_2_ (Fig. 2C) shows that diffraction peaks were strong and sharp, confirming the good crystallinity of the sample. The XRD pattern of MIL-125-NH_2_ shows peaks at 2θ angles of 6.8°, 9.8°, 11.7°, 16.5°, 6°, and 19.3°, corresponding to the (101), (200), (211), (222), and (400) crystal faces of MIL-125-NH_2_, which indicate the formation of MIL-125-NH_2_ (Abdelhameed and El-Shahat 2023). The FTIR of MIL-125-NH_2_ (Fig. 2D) showed two peaks appearing at 3500 and 3380 cm^−1^, which are associated with the amino group (− NH_2_) in MIL-125-NH_2_. The figure exhibited typical vibrational peaks in the region of 1400–1700 cm^−1^ which are associated with the carboxylic acid functional group of the Ti-coordinated MOF structure. A peak appeared at 1630 cm^−1^ which is assigned to the stretching vibration of the N–C bond. A peak appeared at 1580 cm^−1^ which is assigned to the carbonyl group (C = O) asymmetric stretching vibrations. A peak appeared at 1410 which is assigned to symmetric stretching vibrations of C-O. The peak appeared at 1250 cm^−1^ which is associated with the C–H symmetric stretching vibrations of the benzene ring. The peaks appearing in the region 400–800 cm^−1^ are related to the stretching vibration of Ti–O–Ti (Abdelhameed and El-Shahat 2023).

**Fig. 3:**
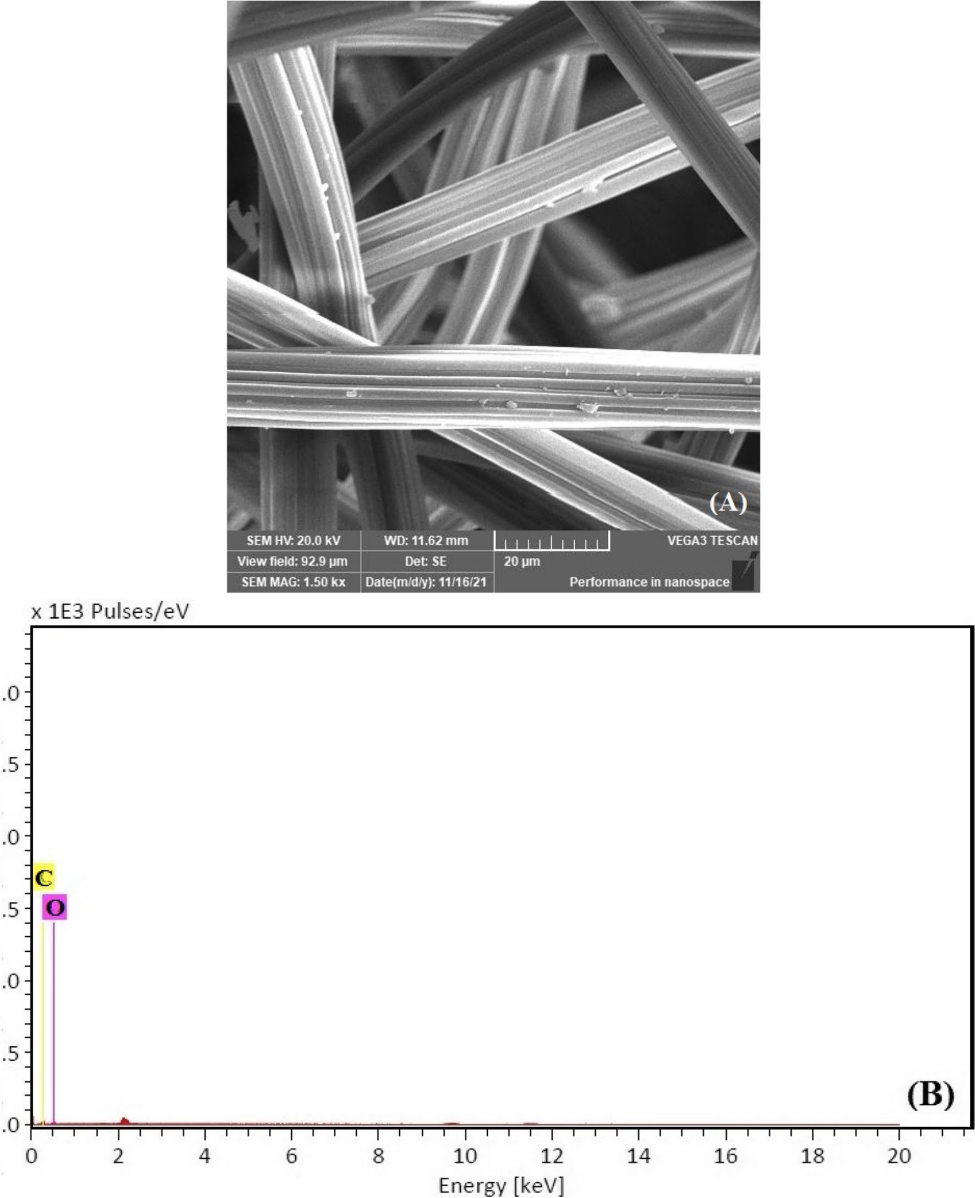

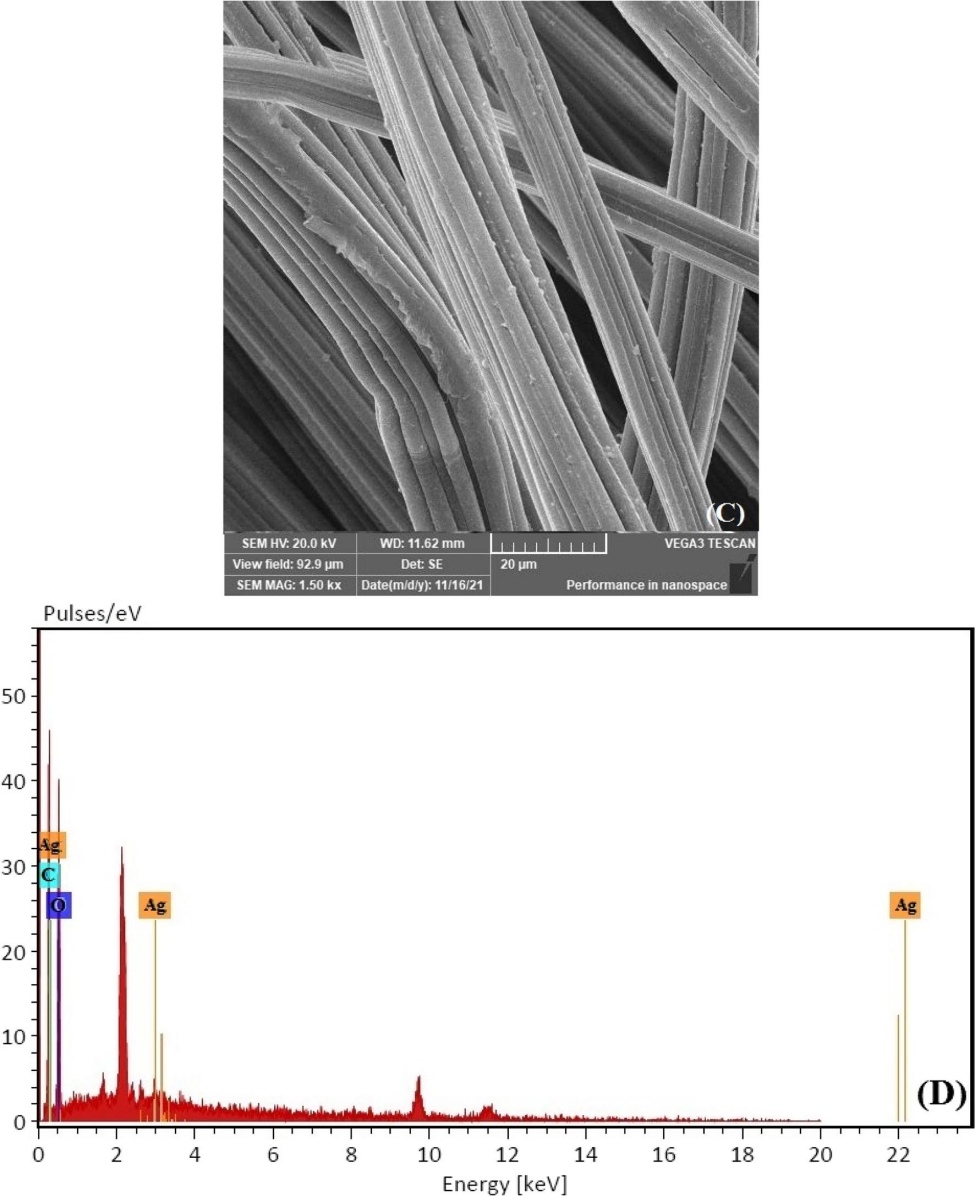

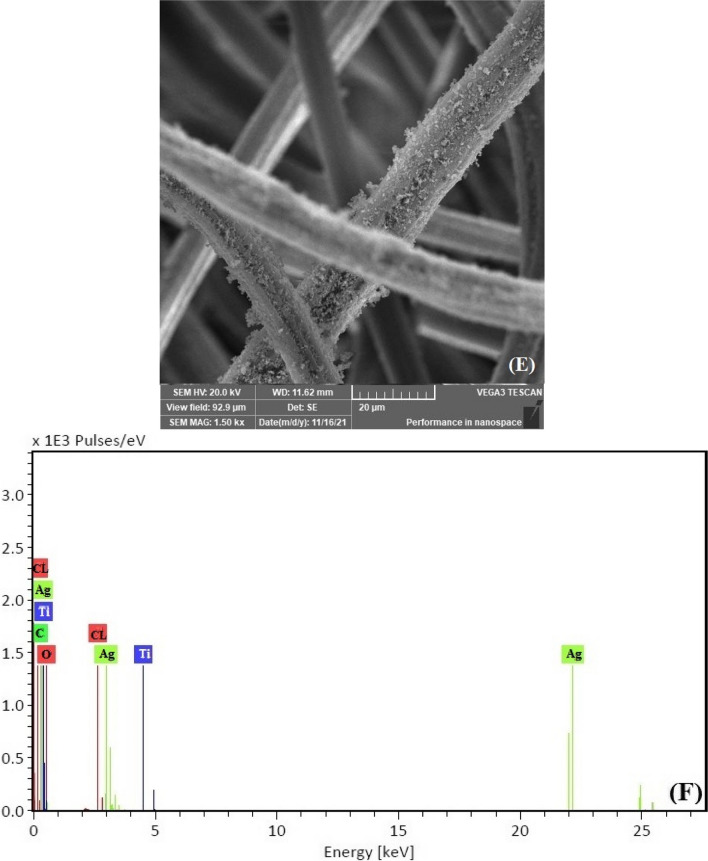

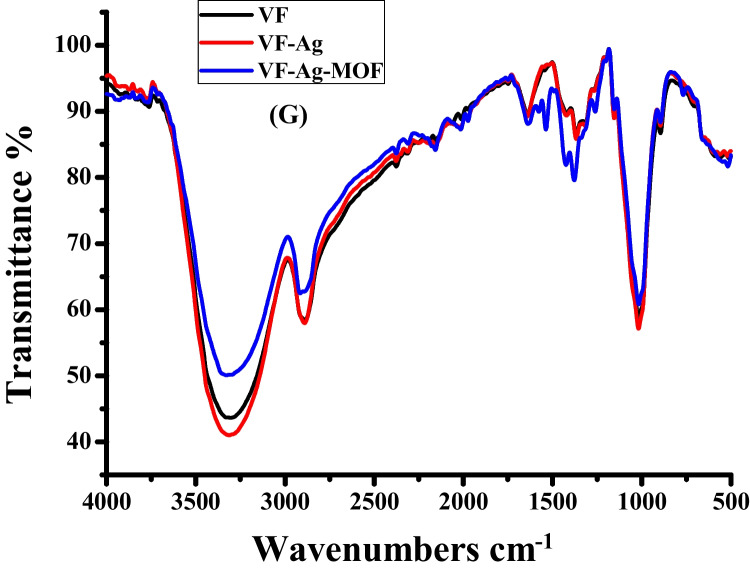
SEM–EDX image for VF (**A**,** B**), VF-Ag (**C**, **D**), VF-Ag-MOF (**E**, **F**), and FTIR of VF-Ag, VF-Ag, and VF-Ag-MOF (**G**)

The surface morphology of the VF, VF-Ag, and VF-Ag-MOF was investigated by scanning electron microscopy (SEM) together with energy-dispersive X-ray spectroscopy (EDX). The VF possesses a surface with a longitudinal fibril structure (Fig. 3A), while the VF-Ag demonstrates that the surface of the VF was coated with uniformly dispersed spherical silver nanoparticles (Fig. 3C). The SEM picture of VF-Ag-MOF (Fig. 3E) reveals that the surface of VF was coated with microsized crystal discs of MOF, although some microporous was detected free after the inclusion of MIL-125-NH_2_. The EDX spectrum of VF (Fig. 3B) revealed two peaks related to carbon and oxygen. The EDX spectrum of VF-Ag (Fig. 3D) revealed a peak at 3 eV caused by Ag L, in addition to the two VF peaks, indicating the existence of silver nanoparticles on the surface of VF. Furthermore, to a signal at 3 eV for silver nanoparticles, the EDX spectrum of VF-Ag-MOF (Fig. 3F) revealed a peak at 4.2 eV, confirming the presence of Ti. Figure 3G shows the FTIR of VF, VF-Ag, and VF-Ag-MOF. The cellulose macromolecule is responsible for the characteristic peaks of VF, which are located at 3480 cm^−1^ attributed to the stretching of O–H, 2910 cm^−1^ attributed to the stretching of C-H, 1660 cm^−1^ attributed to the stretching of C = O, 1515 cm^−1^ attributed to the wagging of C-H, 1405 cm^−1^ attributed to the bending of C-H, and 1210 cm^−1^ attributed to the stretching of C–O. The FTIR spectra of the VF-Ag exhibited no new identifiable peaks, indicating that no substantial chemical reaction on the VF surface occurs during the in situ deposition of Ag (Rehan et al. 2017). In the case of VF-Ag-MOF, two additional peaks were observed at 1575 cm^−1^ and 870 cm^−1^ which are characterized by the O-C-O in the carboxylate of MIL and Ti–O stretching, respectively. In addition, the peak of O–H in VF was shifted to 3450 cm^−1^ (Abdelhameed and El-Shahat 2023).

### Mechanism of decoration of VF with Ag NPs, MOF, and Ag-MOF

Pre-nucleation, nucleation, and growth are the three stages of in situ formation of Ag inserted into VF. Because of their electrostatic interaction with the OH and/or COOH groups, the silver ions (Ag^+^) adsorbed to and dispersed over the surface of the VF during the pre-nucleation stage. Silver ion mobility may be reduced by electrostatic interaction forces, which can also promote the formation of metal nuclei and regulate their growth (Rehan et al. 2015). One of the simplest methods for creating Ag is reduction using TSC. TSC is oxidized to acetone carboxylate, which has an oxidation potential of 0.97 eV and can be used as a reductant in the formation of Ag. TSC plays several roles in the formation of Ag. To begin, TCS forms an Ag^+^@-citrate complex by strongly interacting with silver ions. Second, at high temperatures, TSC becomes more active and can operate as a reducing agent, converting Ag^+^ to Ag^0^ (Patra et al. 2014). TSC reduced the Ag^+^ ions that were attached to the surface of VF during the nucleation stage. TSC oxidized to acetone carboxylate at high temperatures, while Ag^+^ ions were reduced to Ag nanoparticles. In the growth stage, the silver atoms attain saturation; they agglomerate to form nuclei and seed crystals. The seed formed plays a main role in the formation of the spherical shape of nanoparticles. The Ag NPs are eventually attached to the VF surface due to a strong interaction between their surface and the oxygen atoms of OH and COOH in VF (Rehan et al. 2015).

**Scheme 1:**
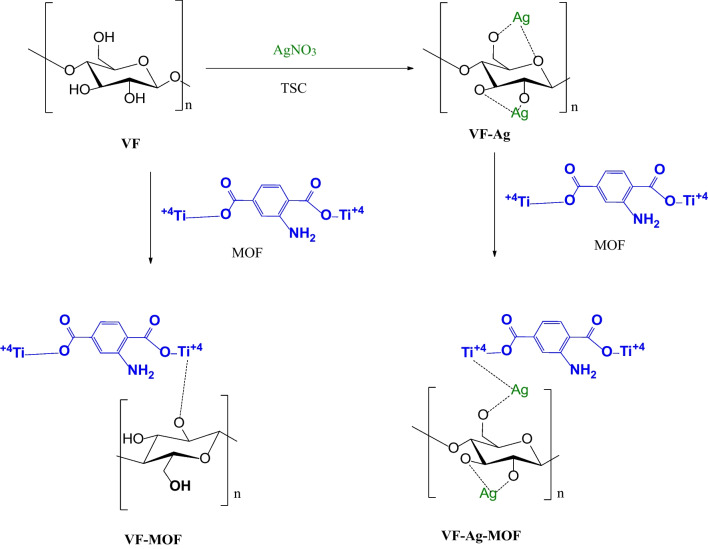
Decoration of the surface of VF with the Ag, MOF, and Ag-MOF

The hydroxyl groups (− OH) in VF combine with the metal center (Ti) of MIL-125-NH_2_ when dispersed MIL-125-NH_2_ in DMF is added to a VF/DMF solution to create VF-MOF. When the scattered MIL-125-NH_2_ in DMF is added to a VF-Ag/DMF solution, the metal center (Ti) of MIL-125-NH_2_ is attached to both the silver and the hydroxyl groups (− OH) in VF to create VF-Ag-MOF (Abdelhameed et al. 2020b). Scheme 1 shows the mechanism of the decoration of the surface of VF with Ag, MOF, and Ag-MOF.

### Sulfa drug removal

Sulfa drugs are typical antibiotics that have been widely used to treat and prevent illnesses like malaria, chlamydia, rheumatic fever, and urinary tract infections (Ovung and Bhattacharyya 2021). The widespread use of sulfa drugs has had a disastrous effect on the aquatic environment. Sulfa drugs are found in different water sources, which pollute the environment and upset the ecosystem’s delicate equilibrium. The urine and feces of humans and animals are considered the main sources of the release of sulfa drugs into the aquatic environment (Baran et al. 2011). It should be mentioned that the majority of sulfa drugs discovered in the environment are in their active forms. They should be effectively eliminated from water due to their potential risks of causing abnormal physiological processes, reproductive impairment, an increase in cancer cases, the development of bacteria resistant to antibiotics, and a potential increase in the toxicity of chemical mixtures (Anuar et al. 2023). Unfortunately, most sulfa drugs are resistant to standard water treatment technologies because of their stable structure and their high polarity due to aromatic rings (Baran et al. 2011; Mulla et al. 2023).

In the current study, the efficiency of VF-Ag-MOF as photocatalytic material was studied by the removal of sulfa drugs through sonocatalytic degradation and sonophotocatalytic degradation.

In the absence of fibers, an initial investigation was conducted to determine the functional significance of ultrasound irradiation (sonolysis) and ultrasound/visible light irradiation (sonophotolysis) in the degradation of sulfa drugs, and the findings are displayed in Table 1. The findings indicated that ultrasound or ultrasound/visible light irradiation caused low sulfa drug degradation within 45 min in the absence of fibers. The degradation of sulfanilamide, sulfadiazine, and sulfamethazine with the sonolysis process was 5, 8, and 10%, respectively. The sonolysis was incapable of sulfa drug degradation due to the significant loss of ^•^OH and H^•^ before attacking the drug molecule. The degradation of sulfanilamide, sulfadiazine, and sulfamethazine with the sonophotolysis process was 9, 11, and 13%, respectively. Because of the more varied reactive species involved in sonophotolysis than in sonolysis, sulfa drug degradation was slightly higher.

**Table 1:** Degradation percentage of sulfa drugs using sonolysis and sonophotolysis

Sulfa drug	Degradation percentage	
	Ultrasound	Ultrasound/visible light
Sulfanilamide	5	9
Sulfadiazine	8	11
Sulfamethazine	10	13

Each value is the mean of triplicate measurements

**Fig. 4:**
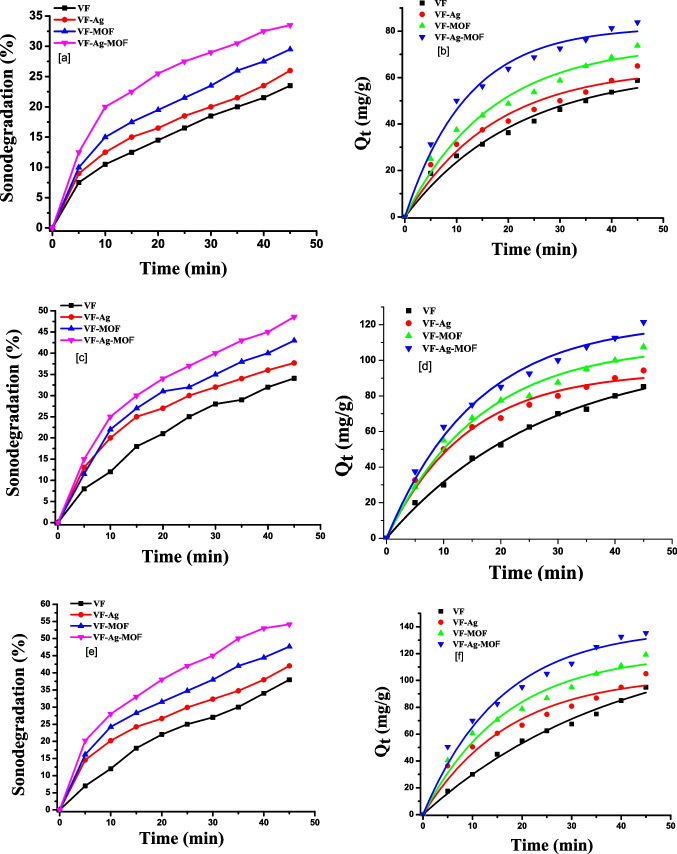
Sonodegradation percentage of sulfanilamide (**a**), sulfadiazine (**c**), or sulfamethazine (**e**) and pseudo-first-order reaction model of sulfanilamide (**b**), sulfadiazine (**d**), or sulfamethazine (**f**). Each value is the mean of triplicate measurements

Figure 4a, c, e shows the degradation percentage of sulfanilamide, sulfadiazine, or sulfamethazine using sonocatalytic degradation in aqueous solutions.

These results show that ultrasound irradiation with different viscose fibers resulted in greater sulfa drug breakdown than ultrasound irradiation without fibers. Because the solid catalysts reduced the solid–liquid tensile strength, the ultrasonic waves generated more cavitation bubbles, which boosted sonoluminescence and water dissociation. Sonoluminescence is the light that is produced by the recombination of free radicals in cavitation microbubbles; this light can also activate catalysis. The turbulence created by sonication also aided mass transfer from the solution to the catalyst surface. The sulfa drugs’ sonocatalytic degradation efficiency increased with increased irradiation time. The sonocatalytic degradation percentage of sulfa drugs was higher in the presence of VF-Ag, VF-MOF, and VF-Ag-MOF than in VF, indicating the effective role of MOF and Ag NPs for the active radical generation in the presence of ultrasound irradiation. The introduction of Ag NPs and MOF into the VF network led to an increase in the sulfa drug degradation efficiency due to the particles being equally distributed over the fibers, creating more active sites to generate more reactive radical species. The sonocatalytic degradation efficiency in the presence of VF-Ag-MOF is higher than that of VF-MOF and VF-Ag. This is explained by the increase in the available surface area and active sites of VF-Ag-MOF, which led to a significant improvement in the adsorption capability of the catalyst for the generation of more reactive radical species as a stronger oxidizing agent.

The sonocatalytic degradation efficiency of the sulfamethazine drug is higher than that of the sulfadiazine drug and the sulfanilamide drug. The sonocatalytic degradation of the sulfanilamide drug was 21, 24, 28, and 34% in the presence of VF, VF-Ag, VF-MOF, and VF-Ag-MOF, respectively, after 45 min (Fig. 4a). The sonocatalytic degradation of the sulfadiazine drug was 31, 36, 42, and 48% in the presence of VF, VF-Ag, VF-MOF, and VF-Ag-MOF, respectively, after 45 min (Fig. 4c). The sonocatalytic degradation of the sulfamethazine drug was 35, 41, 48, and 54% in the presence of VF, VF-Ag, VF-MOF, and VF-Ag-MOF, respectively, after 45 min (Fig. 4e).

Figure 4b, d, f shows the nonlinear kinetic plots of the sonocatalytic degradation amount of the sulfa drug *Q*_*t*_ (mg/g) against time (min). The order of maximal sonodegradation amounts of sulfanilamide (Fig. 4b), sulfadiazine (Fig. 4d), and sulfamethazine (Fig. 4f) is as follows: VF-Ag-MOF (83, 122, and 135 mg/g) > VF-MOF (77, 104, and 120 mg/g) > VF-Ag (62, 90, and 105 mg/g) > VF (56, 82, and 90 mg/g). Table 2 shows the reaction rate constant (*K*_1_), of a pseudo-first-order reaction model, which can be calculated from the nonlinear fit of *Q*_*t*_ (mg/g) against time.

**Table 2:** The coefficient of determination (*R*^2^) and reaction rate constant (*K*_1_)

Samples	Sulfanilamide		Sulfadiazine		Sulfamethazine	
	*K*_1_ (min^−1^)	*R*^2^	*K*_1_ (min^−1^)	*R*^2^	*K*_1_ (min^−1^)	*R*^2^
VF	0.04	0.97	0.03	0.99	0.02	0.99
VF-Ag	0.05	0.96	0.07	0.98	0.05	0.96
VF-MOF	0.06	0.97	0.061	0.98	0.05	0.97
VF-Ag-MOF	0.08	0.98	0.06	0.98	0.06	0.97

Table 2 demonstrated that the coefficient of determination (*R*^2^ ≥ 0.96) values agreed well with the pseudo-first-order kinetic model. The incorporation of Ag and MOF on the surface of VF improved the reaction rate constant (*K*_1_). The degradation rate of sulfa drugs in the presence of VF was lower than in the presence of VF-Ag, VF-VF, and VF-Ag-MOF. The VF-Ag-MOF exhibited the highest rate constant, which was 1.2 times higher than that of the VF. The VF-Ag-MOF has the highest *K*_1_ values of 0.08297, 0.06373, and 0.06375 min^−1^ for sulfanilamide, sulfadiazine, and sulfamethazine.

One significant result of ultrasonic activity on the liquid system is the cavitation effect. A significant number of incredibly tiny cavitation bubbles are produced by the liquid system as a result of the ultrasonic wave’s mechanical energy acting on it. A significant quantity of energy is produced during the process of its production to break, creating regional hot spots and sonoluminescence. A significant number of incredibly tiny cavitation bubbles are produced by the liquid system as a result of the ultrasonic wave’s mechanical energy acting on it. A significant quantity of energy is produced during the process of its production to break, creating regional hot spots and sonoluminescence (Wood et al. 2017). The system’s high pressure and temperature conditions led to the decomposition of H_2_O into HO^•^ and H^+^ radicals. HO^•^ radicals serve as reactive oxygen species to degrade the drug (Abdurahman et al. 2021). Supplementary nuclei increased the rate of H_2_O molecule breakdown and produced more HO^•^ radicals, in addition to improving the ultrasonic degradation performance in the presence of MIL-125-NH_2_ (Ti). This resulted in an acceleration of the creation of microbubbles. Additionally, sonoluminescence, which produces light with a wide variety of wavelengths, can be caused by ultrasonic cavitation (Abdurahman et al. 2021). This light can excite MIL-125-NH_2_ (Ti) to generate electrons and holes. The generated electrons and holes are the main source of reactive oxygen species, which can be used to degrade the drug.

However, ultrasounds alone did not achieve outstanding degradation efficiencies for the sulfa medicines. Thus, coupling ultrasound with visible light irradiation can enhance the degradation efficiency through the sonophotocatalytic process. The sonophotocatalytic process is a hybrid sonocatalytic and photocatalytic process.

**Fig. 5:**
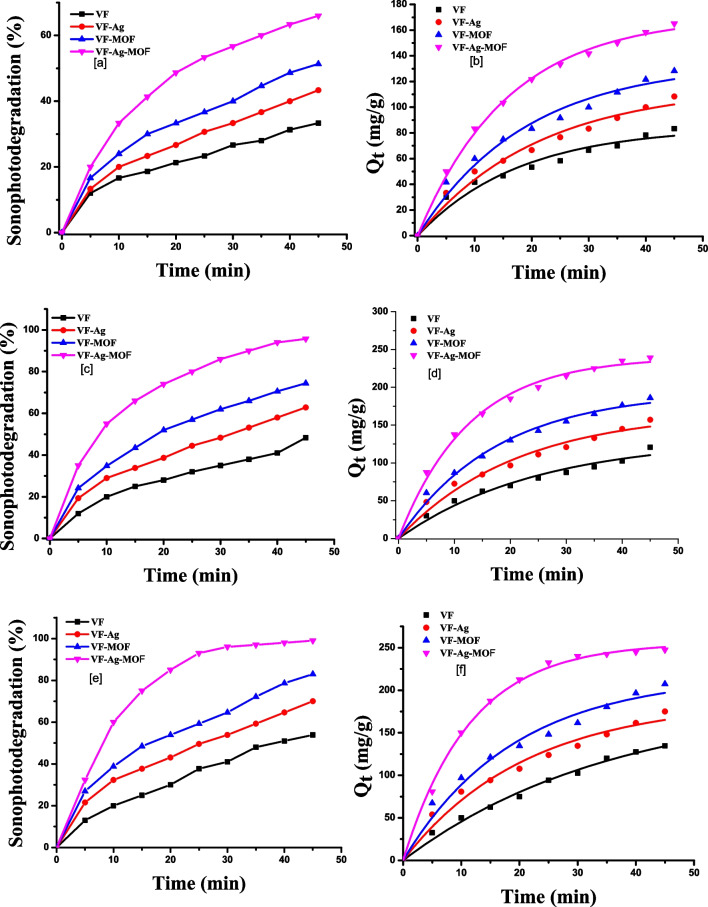
Sonophotodegradation percentage of sulfanilamide (**a**), sulfadiazine (**c**), or sulfamethazine (**e**) and pseudo-first-order reaction model of sulfanilamide (**b**), sulfadiazine (**d**), or sulfamethazine (**f**). Each value is the mean of triplicate measurements

Figure 5a, c, e shows the degradation of sulfanilamide, sulfadiazine, or sulfamethazine using sonophotocatalytic degradation in aqueous solutions.

These figures reveal that the addition of visible light significantly enhances the sonocatalytic performance of the ultrasound system. Combining ultrasound with visible light irradiation increased the efficiency of the degradation of sulfa drugs. The sulfa drug sonophotocatalytic degradation rate increased with increasing ultrasonic and light irradiation times simultaneously. Sonophotodegradation efficiency towards sulfa drugs is significantly higher for VF-Ag-MOF and VF-MOF when compared to sonodegradation efficiency. This is attributed to the photodegradation ability of the Ag-MOF and MOF photocatalysts under visible light. Sulfamethazine has higher sonophotocatalytic degradation efficiency than sulfadiazine and sulfanilamide drugs. A synergistic effect (2.5-fold) was observed for VF-Ag-MOF for the three drugs by combining ultrasound and visible light irradiation. The sonophotocatalytic degradation of the sulfanilamide drug was 29, 34, 50, and 65% in the presence of VF, VF-Ag, VF-MOF, and VF-Ag-MOF, respectively, after 45 min (Fig. 5a). The sonophotocatalytic degradation of the sulfadiazine drug was 40, 58, 65, and 90% in the presence of VF, VF-Ag, VF-MOF, and VF-Ag-MOF, respectively, after 45 min (Fig. 5c). The sonophotocatalytic degradation of the sulfamethazine drug was 42, 63, 83, and 98% in the presence of VF, VF-Ag, VF-MOF, and VF-Ag-MOF, respectively, after 45 min (Fig. 5e).

Several factors can be used to explain the beneficial effects of combining ultrasound with visible light irradiation. (i) ^•^OH generation increased in the reaction combination; (ii) more hydrogen peroxide may be produced in water via photocatalysis and sonolysis, photocatalysis via conduction band electron reduction of adsorbed dioxygen, and sonolysis via recombination between hydroxyl radicals produced by cavitation bubble implosion; (iii) the improvement in the mass transfer of the drug between the liquid phase and catalyst surface; and (iv) increased activity due to the catalyst particles’ increased surface area after being de-aggregated by ultrasound.

Figure 5b, d, f shows the nonlinear kinetic plots of the sonophotocatalytic degradation amount of sulfa drug *Q*_*t*_ (mg/g) against time (min). The order of the highest sonophotodegradation amount of sulfanilamide (Fig. 5b), sulfadiazine (Fig. 5d), and sulfamethazine (Fig. 5f) is as follows: VF-Ag-MOF (165, 230, and 245 mg/g) > VF-MOF (128, 175, and 210 mg/g) > VF-Ag (110, 150, and 170 mg/g) > VF (75, 110, and 135 mg/g).

Table 3 shows the reaction rate constant (*K*_1_) of a pseudo-first-order reaction model, which can be calculated from the nonlinear fit of *Q*_*t*_ (mg/g) against time.

**Table 3:** The coefficient of determination (*R*^2^) and reaction rate constant (*K*_1_)

Samples	Sulfanilamide		Sulfadiazine		Sulfamethazine	
	*K*_1_ (min^−1^)	*R*^2^	*K*_1_ (min^−1^)	*R*^2^	*K*_1_ (min^−1^)	*R*^2^
VF	0.03	0.95	0.04	0.97	0.02	0.99
VF-Ag	0.04	0.97	0.04	0.97	0.04	0.97
VF-MOF	0.05	0.97	0.05	0.99	0.05	0.97
VF-Ag-MOF	0.08	0.99	0.07	0.99	0.08	0.99

Table 3 demonstrated that the coefficient of determination (*R*^2^ ≥ 0.96) values corresponded well to the pseudo-first-order kinetic model. The incorporation of Ag and MOF improved the rate constant (*K*_1_) of a reaction. The degradation rate of sulfa drugs in the presence of VF was lower than in the presence of VF-Ag, VF-VF, and VF-Ag-MOF. The VF-Ag-MOF exhibited the highest rate constant, and it was higher than that of the rate constant of sonophotocatalytic degradation. VF-Ag-MOF has the highest *K*_1_ values of 0.08335, 0.07945, and 0.08678 min^−1^ for sulfanilamide, sulfadiazine, and sulfamethazine.

The reaction mechanism of the drug’s sonophotocatalytic degradation is primarily driven by the synergistic interaction between sonolysis, sonocatalysis, and photocatalysis (Abdurahman et al. 2021). The creation of hot spots (high temperatures and pressures) caused by ultrasonic cavitation and visible light irradiation are the major components that generate the reactive oxygen species needed to degrade the drug (Abdurahman et al. 2021). Ti, as a metal center that serves as a cluster, is coordinated with 2-amino terephthalic acid as an organic carboxylic acid that acts as a ligand to form MIL-125-NH_2_. The band gap of prepared MIL-125-NH_2_ is 2.65 eV with a BET surface area of 1052 m^2^/g (Abdelhameed and El-Shahat 2019). Ti’s empty outer orbitals are the conduction band (CB), while 2-amino terephthalic acid’s outer orbitals are the valence band (VB). Under light irradiation, MIL-125-NH_2_ is activated producing electron–hole pairs (Emam et al. 2019). Electrons (e^−^) are excited from the VB to the CB, causing holes (h^+^) to form in the VB. When holes (h^+^) react with H_2_O or − OH, the HO^•^ radicals are formed, which function as reactive oxygen species to degrade the drug. Electrons (e) are captured by dissolved O_2_ to create O_2_^•−^ radicals, which also serve as reactive oxygen species to degrade the drug (Zhang et al. 2013). The photocatalytic activity of MIL-125-NH_2_ is enhanced by Ag NPs. In the presence of Ag NPs, the electrons (e^−^) in the CB of MIL-125-NH_2_ are expected to travel swiftly to the VB of the Ag NPs because of the production of a Schottky barrier at the junction between MIL-125-NH_2_ and Ag NPs. Moreover, the Ag NPs operate as a suitable separation barrier between the holes and electrons, preventing their recombination, which leads to improved photocatalytic activity of MIL-125-NH_2_ (Seery et al. 2007). Also, Ag NPs aid electron excitation by forming an electric field, and the LSPR effect of Ag NPs augments this electric field (Hou and Cronin 2013). Thus, the combination of light irradiation and ultrasonic irradiation can accelerate the degradation rate of the drug through the increased generation of reactive oxygen species. The active radicals produced by photocatalysis interact with the drug that has been adsorbed on the MOF’s surface. The subsequent desorption of the drug from the MOF surface by the shock waves created by the cavitation bubbles takes place. Additionally, the radicals created by the momentary implosion of cavitation bubbles around the MOF may contribute to the drug’s degradation (Abdurahman et al. 2021; Emam et al. 2019).

### Evaluation of catalytic activity

Nitroaromatic compounds are a class of organic compounds that contain at least one nitro group (− NO_2_) on the benzene ring. Numerous applications, including the production of explosives, polymers, dyes, insecticides, and other intermediates, rely heavily on nitroaromatic compounds. The release and improper disposal of these nitroaromatic compounds from these industries into the water cause serious problems in the environment and water system (Xia et al. 2023). Nitroaromatic compounds pose a risk to human health and aquatic life due to their high toxicity, mutagenic, carcinogenic potential, and non-biodegradability (Kovacic and Somanathan 2014). The reduction of nitroaromatic compounds can decrease their toxic and harmful effects and improve their degradation rate in the environment. Nitrophenol compounds are the most common type of nitroaromatic compounds. The p-nitrophenol (4-NP) is one of the significant causes of water pollution, but its derivative, p-aminophenol (4-AP), is exceptionally beneficial to various industries (Karimi-Maleh et al. 2023). The conversion of 4-NP to 4-AP is considered the most efficient, low-cost, and environmentally friendly process. The conversion of 4-NP to 4-AP requires the use of reducing agents such as sodium borohydride (NaBH_4_). The thermodynamic prospects for the reduction of 4-NP by NaBH_4_ (E_0_ (H_3_BO_3_/BH_4_^−^) = 1.33 V) are fairly favorable (E^0^ (4-NP/4-AP) = ** − **0.76 V). Despite this, because of the considerable difference in the reduction potentials of NaBH_4_ and 4-NP, this interaction was not kinetically reached in the absence of a catalyst (Abdelhameed and El-Shahat 2019, Din et al. 2020; Ehsani et al. 2023; Nguyen et al. 2022). The noble metal nanoparticles as a catalyst are widely used in the conversion of 4-NP to 4-AP along with NaBH_4_.

**Fig. 6:**
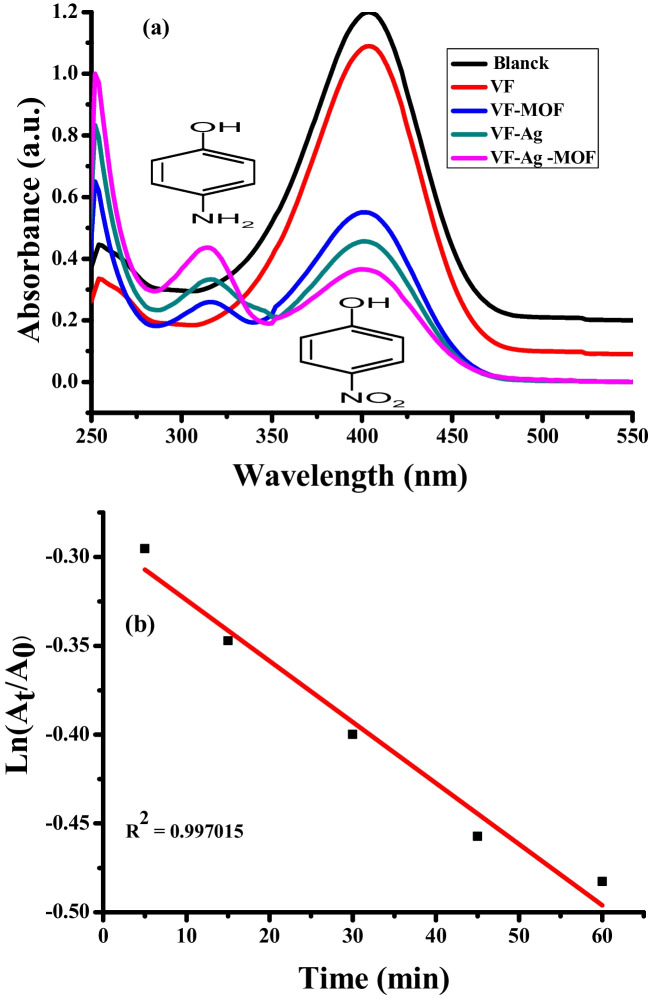
UV–vis absorption spectra of the 4-NP to 4-AP conversion (**a**) kinetics of the catalytic performance for VF-Ag-MOF (**b**)

In this work, the outstanding catalytic performance of VF-Ag-MOF was evaluated through a reduction reaction of 4-nitrophenol in the presence of NaBH_4_. When 4-nitrophenolate ions are produced in the presence of NaBH4, 4-NP absorbs at 400 nm, allowing the reduction process to be tracked. In contrast, 4-AP absorbs at 320 nm due to the electrical n–π* transition state generated by the lone pair of electrons on the oxygen and nitrogen atoms in the compound’s molecular structure. Figure 6a shows the UV–vis absorption spectrum of the catalytic reduction reaction of VF, VF-Ag, VF-MOF, and VF-Ag-MOF for the conversion of 4-NP to 4-AP in the presence of NaBH4 after 60 min. This figure demonstrates that VF marginally changed the absorption peak at 400 nm, indicating a lack of a role for NaBH_4_ to completely convert 4-NP to 4-AP within 60 min. This outcome demonstrates that VF lacks catalytic activity. In the presence of MOF or Ag NPs incorporated into the VF, the absorption peak at 400 nm decreases, while the strength of the new absorption peak at 320 increases, indicating the conversion of 4-NP to 4-AP. VF-Ag-MOF showed the highest catalytic activity, followed by VF-Ag and VF-MOF. The abundance of catalytic active sites like Ag and MOF on the surface of VF may be responsible for the increase in its catalytic activity. The conversion percentage of 4-NP to 4-AP was 9, 54, 62, and 69% for VF, VF-MOF, VF-Ag, and VF-Ag NPs-MOF at 60 min, respectively. Thus, VF-Ag-MOF demonstrated catalytic and convenient separation properties, which may be conveniently recycled by extracting them from the solution with tweezers. The rate constant of the reduction reaction of the conversion of 4-NP in the presence of VF-Ag-MOF was calculated from the plot of Ln (*A*_*t*_/*A*_0_) vs. time (Fig. 6b). The calculated value of 0.00341 s^−1^ at room temperature and the linear coefficient (*R*^2^) value of 0.997015 confirm the pseudo-first-order kinetics of the reduction reaction.

NaBH_4_ acted as the hydrogen source and electron donor in this catalytic process, whereas 4-NP acted as the electron acceptor adsorbed on the surface of MOF or Ag NPs that were already incorporated into the VF surface. As a result, Ag or MOF acted as intermediaries for an electron-transfer mediator of electrons from the NaBH_4_ ion to 4-NP, thereby accelerating the catalytic reaction (Ehsani et al. 2023; Hu et al. 2022; Sharma et al. 2022).

### MB dye removal

Synthetic dyes and colorants are considered the major contributors to water pollution. Synthetic dyes are complex aromatic compounds, and they are widely used in a range of industries, including paper, plastic, food, cosmetics, pharmaceuticals, printing, leather, and textiles (Murtaza et al. 2022). Azo dyes are among the most frequent, and they are widely utilized in different industries because of their simple synthesis process as well as their relatively inexpensive. The majority of these synthetic dyes are typically discarded into the water system once they have exhausted their purpose. The textile industry is a major source of dyes released into the water system (Sharma et al. 2021). Synthetic dyes are highly toxic and carcinogenic, as are their transformation products, and have low biodegradability. Therefore, they pose serious risks to human and aquatic life. Because of their high water solubility, conventional procedures struggle to adequately eliminate them, making them difficult to remove (Varjani et al. 2020).

**Fig. 7:**
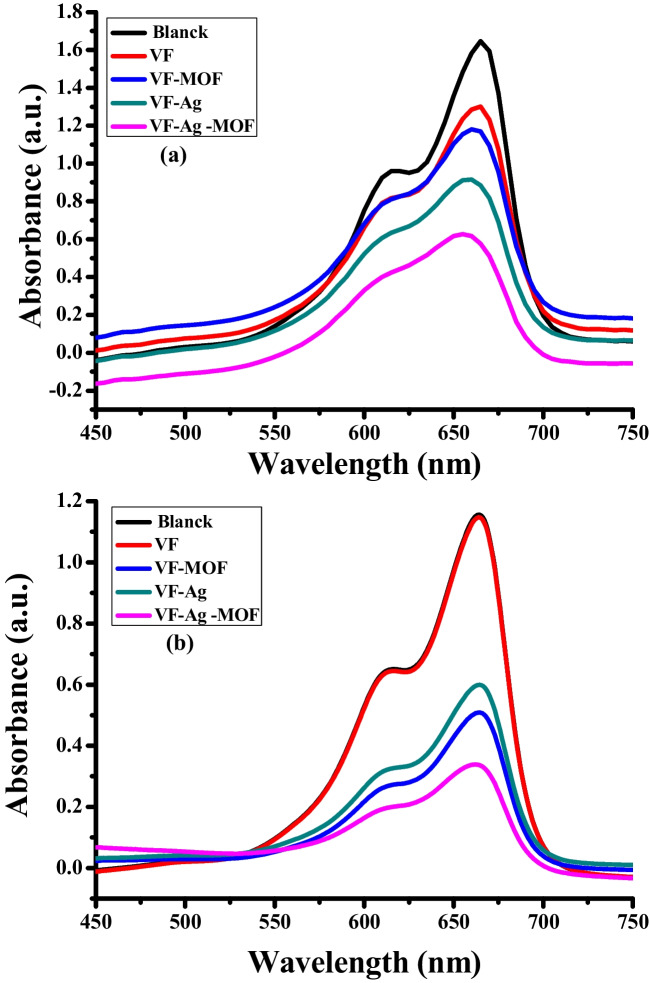
UV–vis absorption spectra of reduction (**a**) photodegradation (**b**) of MB dye

The efficiency of VF, VF-Ag, VF-MOF, and VF-Ag-MOF in the removal of MB dye through catalytic or photogeneration activities was evaluated. Figure 7a, b shows UV–vis spectra of the reduction or photodegradation of MB dye with different samples, respectively. Table 4 shows the reduction percentage or photodegradation percentage of MB dye.

**Table 4:** The reduction percentage or photodegradation percentage of MB dye

Sample	Reduction percentage	Photodegradation percentage
VF	21 ± 2	1 ± 0.1
VF-MOF	29 ± 2	56 ± 2
VF-Ag	46 ± 1	48 ± 1
VF-Ag-MOF	65 ± 2	71 ± 2

Each value is the mean of triplicate measurements

The results of the catalytic experiment reveal (Fig. 7a) that the reduction rate of MB was significantly slower in the absence of a catalyst (VF sample), in which a 21% reduction in 60 min was observed. In the presence of MOF, Ag, and Ag-MOF, the reduction rate of MB increased to 29%, 46%, and 65% at 60 min. The enhancement of the reduction rate of MB on the surface of VF-MOF is due to the high surface area of MOF (1052 m^2^/g), which adsorbed more MB on the VF surface, leading to the increased effect of NaBH_4_ in the reduction process. The enhancement of the reduction rate of MB on the surface in the presence of Ag is due to Ag acting as an active site and acting as a mediator for the electron transfer step during the reduction of MB by NaBH_4_. VF-Ag-MOF is the highest sample in reduction percentage because of the integration between the high surface area of MOF and the role of silver in the reduction process.

The results of the photodegradation experiment reveal (Fig. 7b) that the photodegradation rate of MB in contact with VF was neglected (1%), indicating the VF itself does not possess any photocatalytic activity In the presence of MOF, Ag, and Ag-MOF, the photodegradation rate enhanced. VF-Ag sample showed a photodegradation rate of 48%, which is attributed to the Ag showing good, highly efficient, and stable photocatalysts under visible light irradiation. In the presence of MOF, and Ag-MOF, on the VF surface, the reduction rate of MB increased to 56% and 65% in 60 min. The high photodegradation rate of the VF-Ag-MOF sample confirms the synergetic effect between Ag and MOF in photodegradation under visible light irradiation (Che et al. 2023; Liu et al. 2021; Mukherjee et al. 2022).

The results of both experiments reveal that the photodegradation process is more efficient in the removal of MB dye than in the reduction process. The catalytic removal of MB by the VF-Ag sample followed the reductive pathway as well as the photocatalytic route at almost the same rate.

## Conclusion

In the current study, a cost-effective strategy was designed by the integration of the photocatalytic properties of MIL-125-NH_2_ and the catalytic properties of Ag NPs supported on the surface of viscose fibers (VF). The strategy involved two steps. (i) The first step is to decorate the VF surface with the in situ synthesis of Ag NPs using TSC as a reducing agent to create VF-Ag. The second step is to decorate the VF and V–Ag surface with the titanium metal–organic framework MIL-125-NH_2_ (MOF) to create VF-MOF and VF-Ag-MOF.

The efficiency of VF VF-Ag, VF-MOF, and VF-Ag-MOF was evaluated in challenging environmental applications by studying the removal of different organic contaminations.

(i) Sonocatalytic degradation and sonophotocatalytic degradation of sulfa drugs including sulfanilamide, sulfadiazine, and sulfamethazine drugs.
(ii) The catalytic conversion of 4-NP to 4-AP.
(iii) The catalytic reduction and photodegradation of MB dye.

The results show that VF-Ag-MOF exhibited excellent degradation of the sulfa drug through sonocatalytic and sonophotocatalytic processes. The sonophotodegradation efficiency of sulfa drugs is dramatically increased compared to sonodegradation efficiency. The sonocatalytic degradation percentage of sulfanilamide, sulfadiazine, and sulfamethazine drugs in the presence of VF-Ag-MOF was 34, 48, and 54, in comparison to the presence of VF (21, 31, and 35) after 45 min of ultrasonic. The sonophotodegradation degradation percentage of sulfanilamide, sulfadiazine, and sulfamethazine drugs in the presence of VF-Ag-MOF was 65, 90, and 95 after 45 min of ultrasonic and visible light irradiation. In the catalytic activity in the conversion of 4-NP to 4-AP, the VF-Ag-MOF displayed remarkable catalytic activity. The 4-NP to 4-AP conversion percentage was 69 for VF-Ag-MOF in comparison to 9 for VF. In the MB dye removal, the VF-Ag-MOF showed a reduction rate of MB (65%) and photodegradation rate (71%) in comparison to 21% and 1% for VF.

This study demonstrated that the integration of the plasmonic effect of noble nanoparticles (Ag) with the metal–organic framework (MIL-125-NH_2_) immobilized on the substrate surface (VF) can create VF-Ag-MOF, which has excellent sonophotocatalytic, catalytic, and photocatalytic activities and can be easily recycled by removing it from the solution using tweezers. The photocatalytic properties of MIL-125-NH_2_ can be enhanced by doping with silver nanoparticles. The silver nanoparticles can enhance and improve the photocatalytic under visible light irradiation. Moreover, the silver nanoparticles show excellent catalytic activities. The prepared VF-Ag-MOF was characterized by its effectiveness and ease of application in the removal of different organic pollutants. Additionally, VF-Ag-MOF was also distinguished by its uniqueness, ease of use, low maintenance, and broad applicability.

## Data Availability

All data generated or analyzed during this study are included in this published article.
